# Synthesis of Bis-ureas from Bis(*o*-nitrophenyl) Carbonate

**DOI:** 10.3390/molecules13123192

**Published:** 2008-12-15

**Authors:** Maria-Cristina Turoczi, Monika Simon, Valentin Badea, Carol Csunderlik

**Affiliations:** “ POLITEHNICA” University of Timisoara, Department of Applied Chemistry and Engineering of Organic and Natural Compounds, 300006 Timisoara, Piata Victoriei 2, Romania

**Keywords:** Bis(*o*-nitrophenyl) carbonate, Carbamate, Bis-urea

## Abstract

A general method for the preparation of bis-ureas from bis(*o*-nitrophenyl) carbonate has been developed. Directional urea synthesis is achieved by sequential amine addition to bis(*o*-nitrophenyl) carbonate in two steps: in the first step bis(*o*-nitrophenyl) carbonate is reacted with benzylamine to form benzyl-*o*-nitrophenyl carbamate; in the second step the carbamate is reacted with a variety of diamines in toluene to yield bis-ureas.

## Introduction

Bis-urea compounds are well-suited for material science. Bis-urea grafted molecules or polymers give rise to strong hydrogen bonding interactions that have been used to obtain gelling agents [1,2,3,4,5], to structure inorganic materials [6] and also in polymeric assemblies [7, 8]. Also, it has been demonstrated that bis-ureas are guest-host molecules that exhibits molecular recognition [7]. Recently, it has been reported some chiral bis-ureas which can form microfibrillar foams [9] or lyotropic liquid crystals [10].

The reaction of amines or diamines with isocyanates [1, 2, 6] or diisocyanate [4, 5, 9,10,11] is the key step in the preparation of bis-urea. This method is very inconvenient because isocyanates and diisocyanates are usually prepared from phosgene, a very dangerous reagent. Our attention was therefore directed towards bis(*o*-nitrophenyl) carbonate (**1**), a mild reagent, that can be used in organic synthesis instead of phosgene or its derivatives. We have already reported its reactivity in reaction with primary and secondary amines and have obtained successfully di- and trisubstituted ureas [12, 13]. In this paper we wish to report the use of bis(*o*-nitrophenyl) carbonate (**1**) in the synthesis of bis-ureas **3.**

## Results and Discussion

**Scheme 1:**
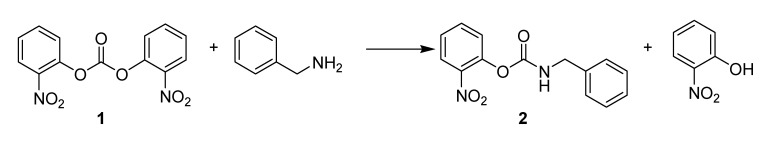
Synthesis of *N*-benzyl-*o*-nitrophenyl carbamate from bis(*o*-nitrophenyl) carbonate.

**Scheme 2:**
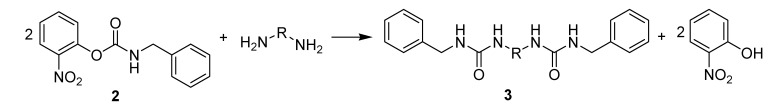
Synthesis of bis-ureas from *N*-benzyl-*o*-nitrophenyl carbamate

An efficient and strainghtforward two-step synthetic route for bis-ureas **3** using bis(*o*-nitrophenyl) carbonate (**1**) has been developed. In the first step, bis(*o*-nitrophenyl) carbonate (**1**) is reacted with benzylamine to produce the intermediate, *N*-benzyl-*o*-nitrophenyl carbamate (**2**, Scheme 1). The second step is the formation of bis-ureas **3** by treatment of this intermediate with various diamines (Scheme 2).

The synthesis of *N*-benzyl-*o*-nitrophenyl carbamate (**2**) was based on the published procedure for the preparation of *o*-nitrophenyl carbamates [14], but without the isolation of this intermediate. Thus to obtain bis-ureas **3** a solution of carbamate **2** which also contains *o*-nitrophenol was treated with a diamine in the molar ratio carbamate: diamine = 2.5:1. The reactions we carried out in toluene because in the second step the transformation of carbamate occurs more slowly and it was necessary to increase the reaction temperature to reflux.

Following our protocol with aliphatic diamine isolation of bis-ureas **3a-f **was achieved, from the reaction mixture by precipitation after one hour, in yields between 86-95% (Table 1). From *o*-phenylenediamine, an aromatic diamine, using 0.1 equiv. of 4-dimethylaminopyridine, bis(benzylcarbamoyl)-*o*-phenylenediamine (**3g**) was obtained in 76%, after two hours of heating.

We also were investigated the reactions with secondary diamines. The bis-urea bis(benzylcarbamoyl)piperazine (**3h**) was easily prepared in high yield by reaction of piperazine with *N*-benzyl-*o*-nitrophenyl carbamate.

**Table 1:** Preparation of bis-ureas from different diamines.

Diamine	Yield (%)	mp (°C)	ν _C=O_ (cm^-1^)	Bis-urea 3
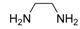	86	240-242	1621	**3a**
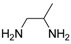	95	228-230	1623	**3b**
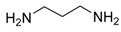	86	217-219	1621	**3c**
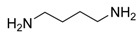	92	245-247	1618	**3d**
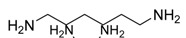	90	224-226	1618	**3e**
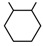	91	255-257	1618	**3f**
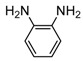	76	197-199	1629	**3g**
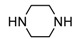	95	190-192	1620	**3h**

## Conclusions

We have developed a convenient two-step synthesis procedure for the preparation of bis-ureas using bis(*o*-nitrophenyl) carbonate as a starting material.

## Experimental

### General

Melting points were determined in a Boetius apparatus (Carl Zeiss Jena). The IR spectra were recorded in KBr pellet for solid compounds and the reaction monitoring was carried out in thermostated silicon cells of 0.137 mm thickness on a Jasco FT/IR-430 instrument. TLC analyses were carried out on pre-coated plates silica gel 60 F_254 _ (Merk). Spot visualization was achieved by exposing the plates under a UV 254 lamp and by treatment with 5% ethanolic phosphomolybdenic acid solution and heating of the dried plates. The ^1^H-NMR and ^13^C-NMR were recorded on a Bruker DPX at 200 MHz in DMSO-*d6*, with TMS as reference. The values of coupling constants are normal for vicinal couplings (CH-CH, CH-NH): 6.5 -7 Hz. Detailed IR, ^1^H-NMR and ^13^C-NMR spectra are available from the authors. Elemental analysis was carried out on a Vario EL instrument. Bis(*o*-nitrophenyl) carbonate (**1**) was obtained by our published method [15, 16]. All reagents were purchased from chemical suppliers and used without further purification.

### General procedure for the preparation of bis-ureas

Compound **1** (200 mg, 0.657 mmol) was dissolved in toluene (10 mL) and benzylamine (1.2 equiv.) was added. The reaction mixture was stirred at room temperature until no further carbonate could be detected by TLC (eluent: dichloromethane). The reaction mixture was washed with 1M HCl solution (5 mL), dried over anhydrous Na_2_SO_4_ and filtered. The diamine (2.5 equiv.) was then added to the filtrate and the resulting reaction mixture was heated under reflux conditions until no diamine was detectable by TLC (eluent: dichloromethane). The insoluble bis-urea **3** obtained was filtered off and washed with cold toluene and a recrystalization from ethanol was performed.

*Bis(benzylcarbamoyl)-ethylenediamine* (**3a**). 73.6 mg ( 86%); mp: 240-242 ^o^C; IR (KBr, cm^-1^): 3330, 3031, 1621, 1575, 1264, 697. ^1^H-NMR δ 3.11 (t, 4H), 4.29 (d, 4H), 5.98 (NH, 2H), 6.39 (NH, 2H), 7.29 (m, 10H); ^13^C-NMR δ 40.31, 42.99, 126.39, 126.98, 128.04, 140.8, 158.21; Elemental Analysis [Calculated (Found)] C_18_H_22_N_4_O_2_: C: 66.24 (66.29); H: 6.79 (6.50); N: 17.17 (16.90).

*Bis(benzylcarbamoyl)-1,2-propylenediamine* (**3b**). 84.2 mg (95%); mp: 228-230 ^o^C; IR (KBr, cm^-1^): 3330, 3031, 1623, 1568, 1260, 694; ^1^H-NMR δ 1.02 (d, 3H), 3.03 (t, 2H), 3.71 (m, 1H), 4.22 (d, 4H), 7.23 (m, 10H), 5.86 (NH, 1H), 6.03 (NH, 1H) 6.32 (NH, 1H), 6.42 (NH, 1H); ^13^C-NMR δ 18.97, 40.61, 45.16, 45.94, 126.42, 126.94, 128.08, 140.8, 158.29. Elemental Analysis [Calculated (Found)] C_19_H_24_N_4_O_2_: C: 67.04 (66.62); H: 7.11 (6.73); N: 16.46 (16.14).

*Bis(benzylcarbamoyl)-1,3-propylenediamine* (**3c**). 76.2 mg (86%); mp: 217-219 ^o^C; IR (KBr, cm^-1^): 3355, 3031, 2919, 2870,1621, 1578, 1287, 695; ^1^H-NMR δ 1.48(m, 2H), 3.05 (q, 4H), 4.22 (d, 4H), 5.94 (NH, 2H), 6.34 (NH, 2H), 7.25 (m, 10H); ^13^C-NMR δ 31.03, 38.23, 42.86, 126.36, 126.87, 128.04, 140.87, 158.11; Elemental Analysis [Calculated (Found)] C_19_H_24_N_4_O_2_: C: 67.04 (66.50); H: 7.11 (7.08); N: 16.46 (16.30).

*Bis(benzylcarbamoyl)-1,4-butylenediamine* (**3d**). 85.3 mg (92%); mp 245-247 ^o^C; IR (KBr, cm^-1^): 3338, 3031, 2939, 2863, 1618, 1573, 1268, 695;^ 1^H-NMR δ 1.39 (m, 4H), 3.05 (q, 4H), 4.23 (d, 4H), 5.86 (NH, 2H), 6.21 (NH, 2H), 7.25 (m, 10H); ^13^C-NMR δ 25.57, 40.03, 42.96, 126.38, 129.94, 128.04, 140.83, 158.1; Elemental Analysis [Calculated (Found)] C_20_H_26_N_4_O_2_: C: 67.77 (67.43); H: 7.39 (7.27); N: 15.81 (15.52).

*Bis(benzylcarbamoyl)-1,6-hexamethylenediamine* (**3e**). 90.2 mg (90%); mp: 224-226 ^o^C; IR (KBr, cm^-1^): 3346, 3031, 2932. 2856, 1618, 1576, 1254, 695. ^1^H-NMR δ 1.30 (m, 4H), 1.39 (m, 4H), 3.01 (q, 4H), 4.22 (d, 4H), 5.81 (NH, 2H), 6.18 (NH, 2H), 7.22 (m, 10H); Elemental Analysis [Calculated (Found)] C_22_H_30_N_4_O_2_: C: 69.08 (68.74); H: 7.91 (7.50); N: 14.65 (14.40).

*Bis(benzylcarbamoyl)-1,2-cyclohexylenediamine* (**3f**). 90.9 mg (91%); mp: 255-257 ^o^C;IR (KBr, cm^-1^): 3346, 3031, 2932, 2856, 1618, 1576, 1254, 695; ^1^H-NMR δ 1.27 (m, 4H), 1.41 (m, 4H), 3.31 (m, 2H), 4.21 (d, 4H), 5.78 (NH, 2H), 6.36 (NH, 2H), 7.25 (m, 10H); ^13^C-NMR δ 24.40, 33.03, 42.96, 53.10, 126.35, 126.46, 126.94, 140.83, 158.08; Elemental Analysis [Calculated (Found)] C_22_H_28_N_4_O_2_: C: 69.45 (69.02); H: 7.42 (7.37); N: 14.73 (14.48).

*Bis(benzylcarbamoyl)-1,2-phenylenediamine* (**3g**). 74.6 mg (76%); mp: 197-199 ^o^C; IR (KBr, cm^-1^): 3294, 3030, 1686, 1629, 1247, 696; ^1^H-NMR δ 4.28 (d, 4H), 7.30 (m, 12H) 7.55 (d, 2H), 6.96 (NH, 2H), 7.90 (NH, 2H); ^13^C-NMR δ 42.97, 123.19, 123.43, 126.6, 127.16, 128.15, 140.17, 155.86; Elemental Analysis [Calculated (Found)] C_22_H_22_N_4_O_2_: C: 70.57 (70.05); H: 5.92 (5.52); N: 14.96 (14.85).

*Bis(benzylcarbamoyl)-piperazine* (**3h**). 92.5 mg (95%); mp: 190-192 ^o^C; IR (KBr, cm^-1^): 3302, 3023, 1320, 1620, 1554, 1262, 694; ^1^H-NMR δ 3.37 (t, 8H), 4.27 (d, 4H), 7.09 (NH, 2H), 7.28 (m, 10H); ^13^C-NMR δ 43.23, 43.44, 126.31, 126.93, 127.97, 140.79, 157.37; Anal. Calc. (C_20_H_24_N_4_O_2_): C: 68.16 (67.88); H: 6.86 (6.59); N: 15.90 (15.72).
